# Inhalation-modulated detection of olfactory BOLD responses in the human brain

**DOI:** 10.3389/fnimg.2023.1260893

**Published:** 2023-12-01

**Authors:** Aino-Lotta I. Alahäivälä, Divesh Thaploo, Simon Wein, Philipp Seidel, Marco Riebel, Thomas Hummel, Jens Volkmar Schwarzbach

**Affiliations:** ^1^Biomedical Imaging Group, Department of Psychiatry and Psychotherapy, University of Regensburg, Regensburg, Germany; ^2^Interdisciplinary Center Smell and Taste, Department of Otorhinolaryngology, Technische Universität Dresden, Dresden, Germany

**Keywords:** olfaction, functional magnetic resonance imaging, breathing-modulated analysis, design efficiency, block designs, habituation, effect size maps

## Abstract

**Introduction:**

In contrast to other sensory domains, detection of primary olfactory processes using functional magnetic resonance imaging has proven to be notably challenging with conventional block designs. This difficulty arises from significant habituation and hemodynamic responses in olfactory areas that do not appear to align with extended boxcar functions convolved with a generic hemodynamic response model. Consequently, some researchers have advocated for a transition to event-related designs, despite their known lower detection power compared to block designs.

**Methods:**

Here, we conducted a block design experiment with 16s of continuous odorant stimulation alternating with 16s of continuous odorless air stimulation in 33 healthy participants. We compared four statistical analyses that relied either on standard block designs (SBD1-2) or on block designs that were modulated by the participants' individual breathing patterns (MBD1-2).

**Results:**

We found that such modulated block designs were comparatively more powerful than standard block designs, despite having a substantially lower design efficiency. Using whole-brain effect size maps, we observed that the right insular and medial aspects of the left piriform cortex exhibited a preference for a breathing-modulated analysis approach.

**Discussion:**

Research in olfaction that necessitates designs with longer-lasting blocks, such as those employed in the investigation of state-dependent processing, will benefit from the breathing-modulated analyses outlined in this study.

## 1 Introduction

The processing of olfactory information is intrinsically linked to the rhythmic process of breathing. We perceive odorants in the environment through two types of olfactory stimulation. In orthonasal stimulation, odorous molecules are transported to the cilia of the olfactory mucosa during inhalation and sniffing. In retronasal stimulation, gaseous molecules, for example, those from foods, reach the mucosa through the nasopharynx as we breathe during mastication or exhalation. From the mucosa, olfactory information is transmitted to the olfactory bulb and cortex (Price, 2004). Thus, both orthonasal and retronasal stimulation lead to discrete breathing-related sampling events that underlie olfaction. Previous electrophysiological studies in rabbits (Adrian, 1950) and humans (Haehner et al., 2011) have demonstrated that there is a greater responsiveness of the olfactory system to orthonasal stimuli during inspiration.

The temporal variability of breathing is a challenge for constructing accurate and precise predictors for estimating the amplitude of the Blood Oxygenation Level-Dependent (BOLD) response (Friston et al., 1994; Lindquist et al., 2009; Huettel, 2012) in task-based functional magnetic resonance imaging (task-fMRI). In other sensory modalities, such as vision, temporal information regarding the expected neural and hemodynamic response to a stimulus can be derived with sufficient accuracy if stimulus delivery and the acquisition of functional images are synchronized. In olfaction, breathing adds a non-stationary physiological source of temporal variability to the signal to be detected. Early olfactory neuroimaging studies employed block designs that ignored respiration (Sobel et al., 1997, 1998; Yang et al., 1997; Yousem et al., 1997), followed by slow event-related designs in which explicit computer-controlled instructions to sniff were synchronized with stimulus delivery and image acquisition (Gottfried et al., 2002; Anderson et al., 2003). Later on, the development of respiration-contingent stimulus delivery (Wang et al., 2014) allowed the measurement of olfactory brain activity synchronized with respiration. Respiration-contingent stimulus delivery yields a clear gain in statistical sensitivity, but it requires real-time processing of the participants' respiratory data and real-time control of stimulus delivery, which may not be available in non-specialized laboratories. Furthermore, explicitly instructing participants to sniff (i.e., inhale) at predetermined times may reduce participant comfort or interfere with other experimental goals, such as inducing certain longer-lasting mental states. Taken together, the burden of real-time control, design efficiency considerations from other stimulus modalities (Friston et al., 1999) that favor block designs, or the researchers' goal to induce longer-lasting mental or emotional states may be the underlying reason for why block designs are still commonly used in fMRI studies aimed at mapping the human olfactory system (Donoshita et al., 2021). Several recent studies that have aimed at increasing the sensitivity of block designs in olfaction have found that using rather brief stimulation periods of 3–6 s substantially increased the sensitivity of olfactory mapping experiments (Georgiopoulos et al., 2018; Schäfer et al., 2019) in designs that do not control for respiration. It has been argued that habituation attenuates olfactory signals in the brain, therefore requiring short stimulation periods with long intermittent pauses (Georgiopoulos et al., 2018; Schäfer et al., 2019), which amounts to slow event-related designs that are known to have low statistical detection power (Friston et al., 1998, 1999).

**Figure 1:**
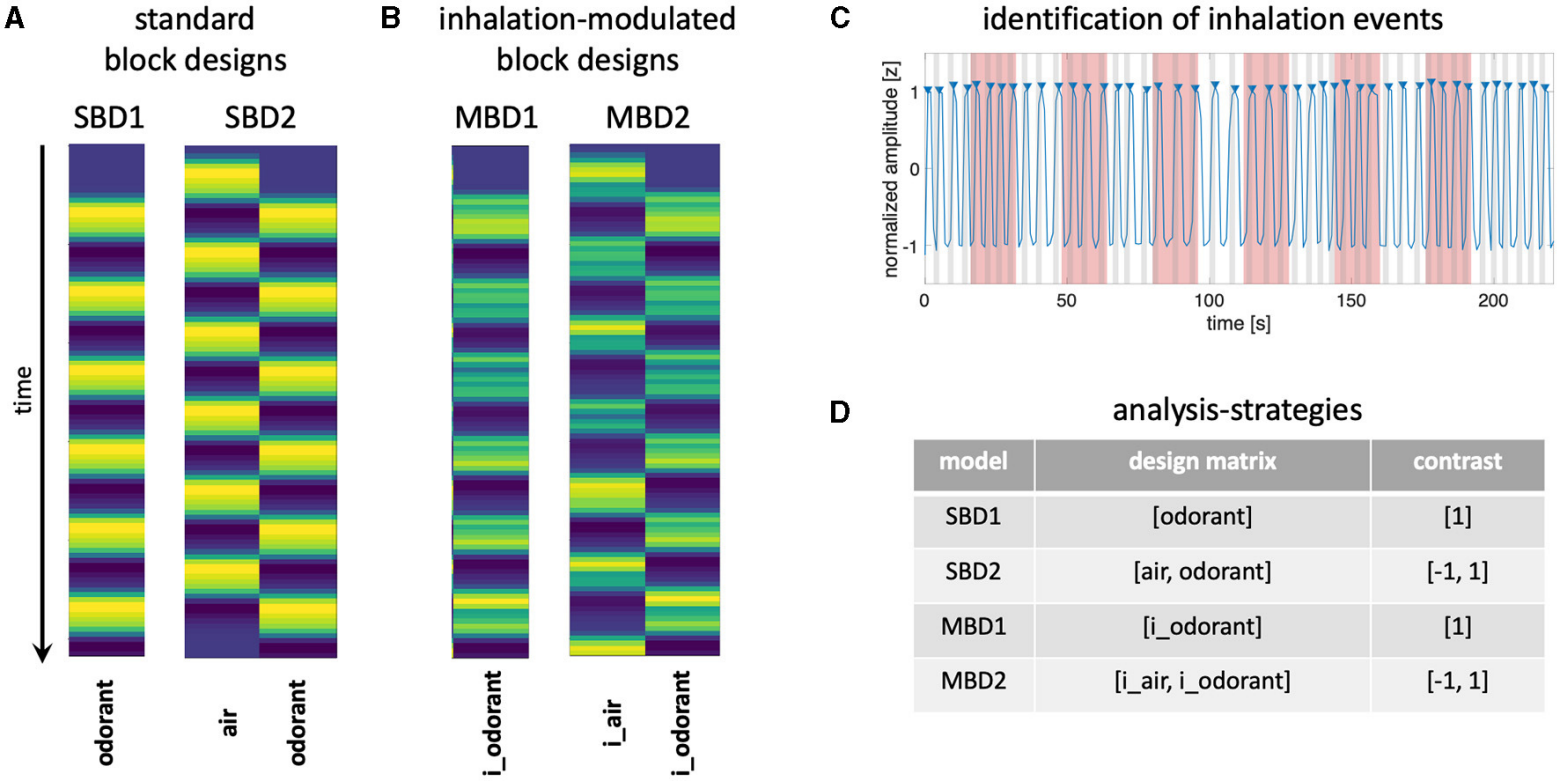
**(A)** The design matrix for the **standard block designs (SBD1-2)** consists of boxcar functions convolved with a hemodynamic response model. The design matrix of SBD1 contains only one predictor for stimulation with an odorant. The design matrix of SBD2 contains two predictors: stimulation with odorless air and stimulation with an odorant. **(B)** Design matrices for **inhalation-modulated block designs (MBD1-2)** are constructed by convolving boxcar functions for stimulation periods with inhalation events (identified in the respiratory signal) and with a hemodynamic response function. MBD1 contains the predictor i_odorant, and MDB2 contains the predictors i_air and i_odorant. **(C)** Normalized amplitude of breathing measured with a breathing belt (blue line). Inhalation events (gray patches) were determined as the 2-s periods that preceded a peak (blue triangles) of the respiratory signal. Blocks during which odorants were presented are shaded in red. Inhalation events were assigned to the condition “i_air” if they fell entirely in the phase in which no odorants were presented. Inhalation events were assigned to the “i_odorant” condition if they fell entirely in the phase in which odorants were presented (red patches). **(D)** We compared four different analysis strategies (SBD1-2 and MBD1-2 for which the table contains the predictors and respective contrasts).

Here, we explore the extent to which brain activity can be efficiently detected in response to longer-lasting experimental blocks by taking into account temporal information about naturally occurring inhalation. To this end, we continuously presented odorants in a block design (16s stimulus on followed by 16s stimulus off) to 33 healthy volunteers who were tasked with breathing normally. We compared two types of standard block designs, representing neural boxcar functions throughout the stimulation periods, convolved with a hemodynamic model with two variants of respiration-modulated analyses (Figure 1), in which we used offline data from a breathing belt to construct respiration-related events in terms of design efficiency and actual detection power.

## 2 Materials and methods

The study was approved by the Institutional Review Board of the University of Regensburg, and all subjects signed an informed consent form. We acquired data from *N* = 33 (19 female) participants.

### 2.1 Data acquisition

All imaging data were acquired on a 3T Siemens Prisma (Siemens Healthcare, Erlangen, Germany) using a 64-channel receiver head coil (Siemens Healthcare) and multiband sequences (epfid2d1_64) provided by the Center for Magnetic Resonance Research (CMRR, Minneapolis, Minnesota, USA). Twelve functional sequences of 104 volumes (total duration 12^*^3 min:28 s) covering the whole brain with 88 slices of 2 × 2 × 2 mm isotropic voxels, repetition time (TR) = 2,000 ms, echo time (TE) = 30 ms, flip angle (FA) = 75°, excitation pulse duration = 9 ms, echo spacing = 0.58 ms, bandwidth = 2,368 Hz/pixel; acquisition matrix (AM) = 96 × 96; field of view (FoV) = 192 × 192 mm; partial Fourier = 7/8 and a multiband acceleration factor of 4 in order to maximize the temporal signal-to-noise ratio (Seidel et al., 2020).

Field map imaging was performed with a double-echo spoiled gradient echo sequence (TR = 715.0 ms, TE = 5.81/8.27 ms, voxel size: 3 × 3 × 3 mm, FA = 40°), which generated a magnitude image and two phase images. The field map image was computed from the two-phase images.

A T1-weighted Magnetization-Prepared Rapid Gradient-Echo (MPRAGE) structural scan was used for co-registration and surface reconstruction (TR = 1,910 ms; TE = 3.67 ms; FA = 9°; FoV = 250 mm^2^; AM = 256 × 256).

Participants were fitted with a stretch-sensitive breathing belt that was wirelessly connected to the scanner's built-in physiological measurement unit (PERU 098, Rev. 10, Siemens, München, Germany). Respiratory data were sampled at 400 Hz.

### 2.2 Design and task

We acquired 12 runs with one odorant per run, consisting of six stimulation blocks of 16s alternating with six blocks without odorant (16s), with each run beginning with 16s without odorant (Figure 1). The order of odorants was pseudorandomized and counterbalanced across participants. Participants were instructed to breathe normally through the nose.

### 2.3 Olfactory stimulation

Odorants (Takasago Europe Perfumery Laboratory S.A.R.L.) and odorless air were presented under computer control at a flow rate of 2.5 L/min using a portable olfactometer (Sommer et al., 2012) that operated outside of the scanning room and which delivered odorized or odorless air through Teflon™ tubes with an inner diameter of 4 mm. In each run, we presented one out of 12 odorants, which were selected to have similar intensities covering a wide range of valences (Table 1).

**Table 1:** Odorants employed in this study.

**Number**	**Odor ID**	**Concentration (%)**	**Valence profile**	**Typical description**
1	Phenyl ethyl alcohol (PEA)	10	Pleasant	Rose
2	Pentadecanolide	10	Pleasant	Fruity
3	Trans-2-hexenyl acetate	10	Pleasant	Citrus
4	4-decanolid	10	Ambivalent	Apricot fruit
5	Citronella	10	Ambivalent	Lemony
6	1-undecanol	10	Ambivalent	Floral
7	Acetophenone	10	Ambivalent	Almond oil
8	Menthyl Isovalerate	100	Ambivalent	Menthol
9	1-benzyl acetate	1	Ambivalent	Gas
10	1-octen-3-ol	10	Unpleasant	Nature
11	Anisole	1	Unpleasant	Anise, fennel
12	3-hexanol	10	Unpleasant	Cut grass

Odorants were diluted with dipropylene glycol.

### 2.4 Processing of respiratory data

Respiratory data were extracted from dicom files using extractCMRRPhysio (https://github.com/CMRR-C2P/MB/blob/master/extractCMRRPhysio.m). We applied a one-dimensional median filter to the respiratory data using Matlab's medfilt1 with an order of 40 (i.e., with a window of 100 ms) before down-sampling the data to a temporal resolution of 1 Hz and finally standardizing (z-transforming) it. Because the physiological monitoring unit of our scanner occasionally recalibrates itself at unpredictable times during a run, there can be large amplitude differences in the breathing data over the course of a few minutes. Since we were interested in the timing of breathing events, i.e., local peaks, but not the amplitudes, we applied an iterative filter (with four iterations) to the filtered and z-transformed respiratory data (y) of each run, with the goal of boosting small signal amplitudes and attenuating large ones:


(1) $$\begin{array}{l} y_{preprocessed}=\frac{y}{|y{|}^{\frac{1}{p}}} \end{array}$$

with *p* = 2 (details on the normalization of respiratory data can be found in Supplementary material S1).

This procedure yielded range-limited (approaching −1 <y <1) transformed respiratory data in with clearly identifiable peaks as shown in Figure 1C for all subjects and runs. We then used Matlab's findpeaks function with a threshold of 0.75 for prominence and a separation of at least 2 s to automatically detect the peaks in the respiratory data. We determined inhalation events as the 2 s that precede each peak and stored this timing information per subject per run.

### 2.5 Processing of imaging data

Functional and structural data were preprocessed with fMRIPrep (Esteban et al., 2019) (version 20.2.4). Preprocessing included automatic segmentation and transformation of T1-weighted images into MNI space (MNI152NLin2009cAsym), bias field correction, motion correction, and slice scan time correction of functional images. The complete, automatically generated description of the fMRIPrep steps can be found in the Supplementary material S2. Additionally, we applied spatial smoothing to the functional images with a Gaussian kernel of 8 mm at FWHM.

Statistical parameter estimation was conducted with custom code in Python that used NI-learn (Abraham et al., 2014) and Matlab code that used functions from CoSMoMVPA (Oosterhof et al., 2016). Forty-eight first-level maps (12 runs × four analysis strategies) were computed per subject.

Since differential processing of different odorants was beyond the scope of this paper, we computed second-level statistics (subject maps) across runs, thereby collapsing results across odorants, leaving 4 s-level maps per subject (SBD1, SBD2, MBD1, and MBD2). Finally, we computed a group-level map (SBD1, SBD2, MBD1, and MBD2) for each analysis strategy.

### 2.6 Design efficiency

We computed the design efficiency (Friston et al., 1999; Liu et al., 2001) for the standard block design and for two versions of the breathing-modulated block design (inhale odorant vs. mean, inhale odorant vs. inhale without odorant)


(2) $$\begin{array}{l} efficiency=\frac{1}{{c\left(X^{T}X\right)}^{-1}c^{T}} \end{array}$$

with (X^T^X)^−1^ is the inverse of the variance-covariance matrix of the design X and c is the contrast vector. Note that for computing efficiency we ignored nuisance variables. Efficiency calculations were performed with custom code in Matlab (version R2021b). A detailed description of how to compute design efficiency can be found at https://lukas-snoek.com/NI-edu/fMRI-introduction/week_3/design_of_experiments.html?highlight=efficiency. In the standard block design (Figure 1A), the design matrix consisted of the predictor **s** for odorant stimulation. The contrast of interest was c_SBD_ = [1]. It should be noted that all design matrices contained a constant term to model the mean, which we have omitted here in our description of the contrast vectors. For the first variant of the breathing-modulated block design MBD1 (“inhale odorant vs. mean”), the design matrix X was constructed as depicted in Figure 1B with the predictors **i_air** and **i_odorant**. The corresponding contrast vector was c_MBD1_ = [1]. The second variant of the breathing-modulated block design MBD2 (“inhale odorant vs. inhale without odorant”) used the same design matrix as MDB1, but a contrast vector c_MBD2_ = [−1 1]. Since we used the same stimulus timing in all blocks, efficiency_SBD_ only had to be computed once. The efficiencies for the breathing-modulated designs MBD1 and MBD2 were computed subject by subject and run by run, and finally averaged across runs, yielding one efficiency score per subject and design variant, respectively.

Voxel-wise effect sizes (Hedge's g) were computed with the Matlab Toolbox “Measures of Effect Size” (version 1.5) (Hentschke and Stüttgen, 2011) for the SBD1-2 and MBD1-2 models. From these four effect size maps, each thresholded at *g* > 0.4, we computed a “winner” map (Figure 4), indicating at each voxel which model yielded the highest effect size.

## 3 Results

Respiration rates did not differ [*t*_(32)_ = 0.508, *p* = 0.615] between blocks without odorants (mean = 14.55 inhalations per min, std = 3.468) and blocks with odorants (mean = 14.47 inhalations per min, std = 3.061).

### 3.1 Statistical parameter maps

**Figure 2:**
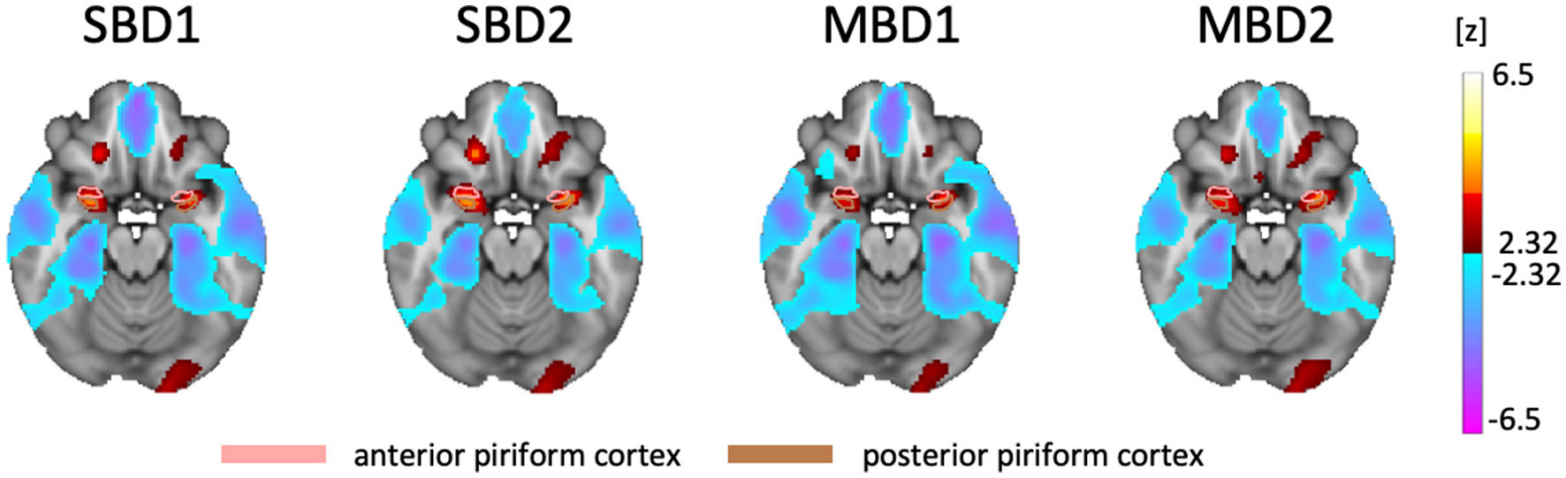
Statistical Parameter Maps for the group analyses of the four compared analysis approaches, all of which yielded bilateral hot spots in piriform and orbitofrontal cortices (FDR-thresholded with *q* < 0.05, *z* = −18). SBD1 and MBD1: positive z values denote BOLD amplitudes above the mean, and negative z values denote BOLD amplitudes below the mean. SBD2 and MBD2: positive values denote that i_odorant—i_air yields positive differences in estimated BOLD amplitudes, while negative values denote that i_odorant—i_air yields negative differences in estimated amplitudes. SBD2 and MBD2 revealed the highest detection power.

Figure 2 shows the group results from the analysis of the standard block designs (SBD1-2) and the inhalation-modulated analyses (MBD1-2) using the respective design matrices and statistical contrasts depicted in Figures 1A, B, D. All four statistical parameter maps contained bilateral clusters of increases in BOLD amplitude in primary olfactory areas, namely the orbitofrontal gyrus (OFG) and the frontal (anterior) and temporal (posterior) piriform cortices.

**Figure 3:**
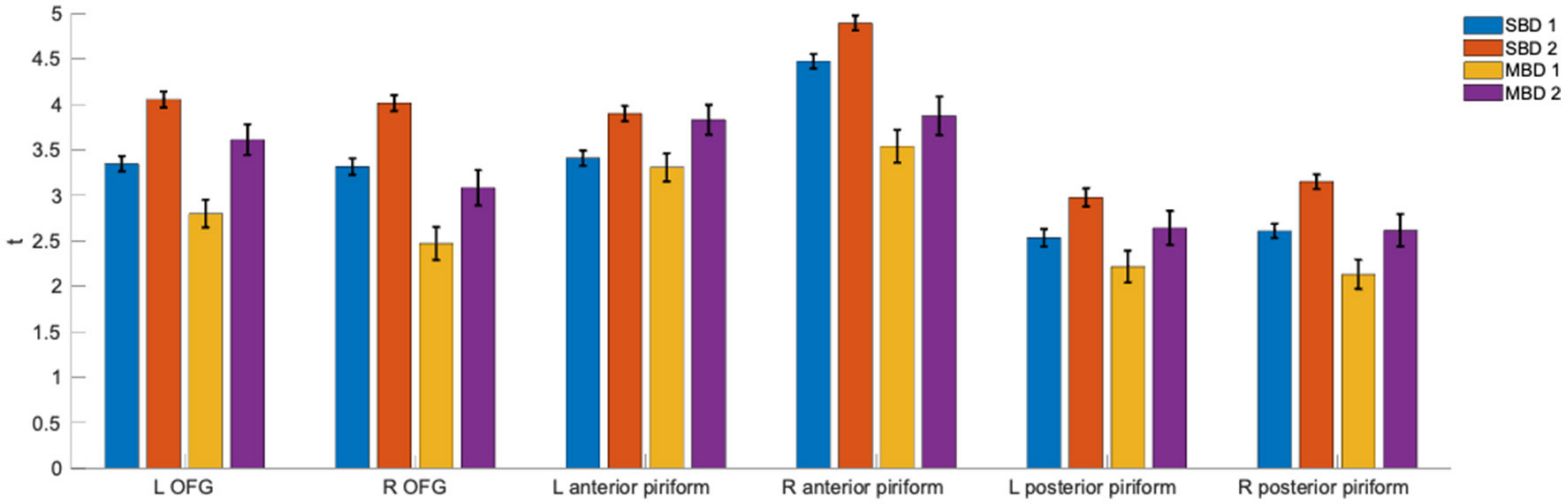
ROI-wise *t*-values and their standard deviations for the statistical contrasts that underlie analyses SBD1-2 and MBD1-2 (the corresponding design matrices and contrasts are found in Figure 1D). Contrasting odorant vs. non-odorant (SBD2 and MDB2) outperformed models that only took stimulation with an odorant (SBD1 and MBD1) into account.

Using regions of interest from an olfactory atlas derived from diffusion imaging (Echevarria-Cooper et al., 2022), we computed statistical parameter estimates of our four analysis strategies in the left and right orbitofrontal gyri (OFG) and anterior and posterior piriform cortices. Figure 3 shows the corresponding *t*-values and their standard deviations. Across all investigated ROIs, it appears that SBD2 yields the strongest effects, followed by MBD2.

**Figure 4:**
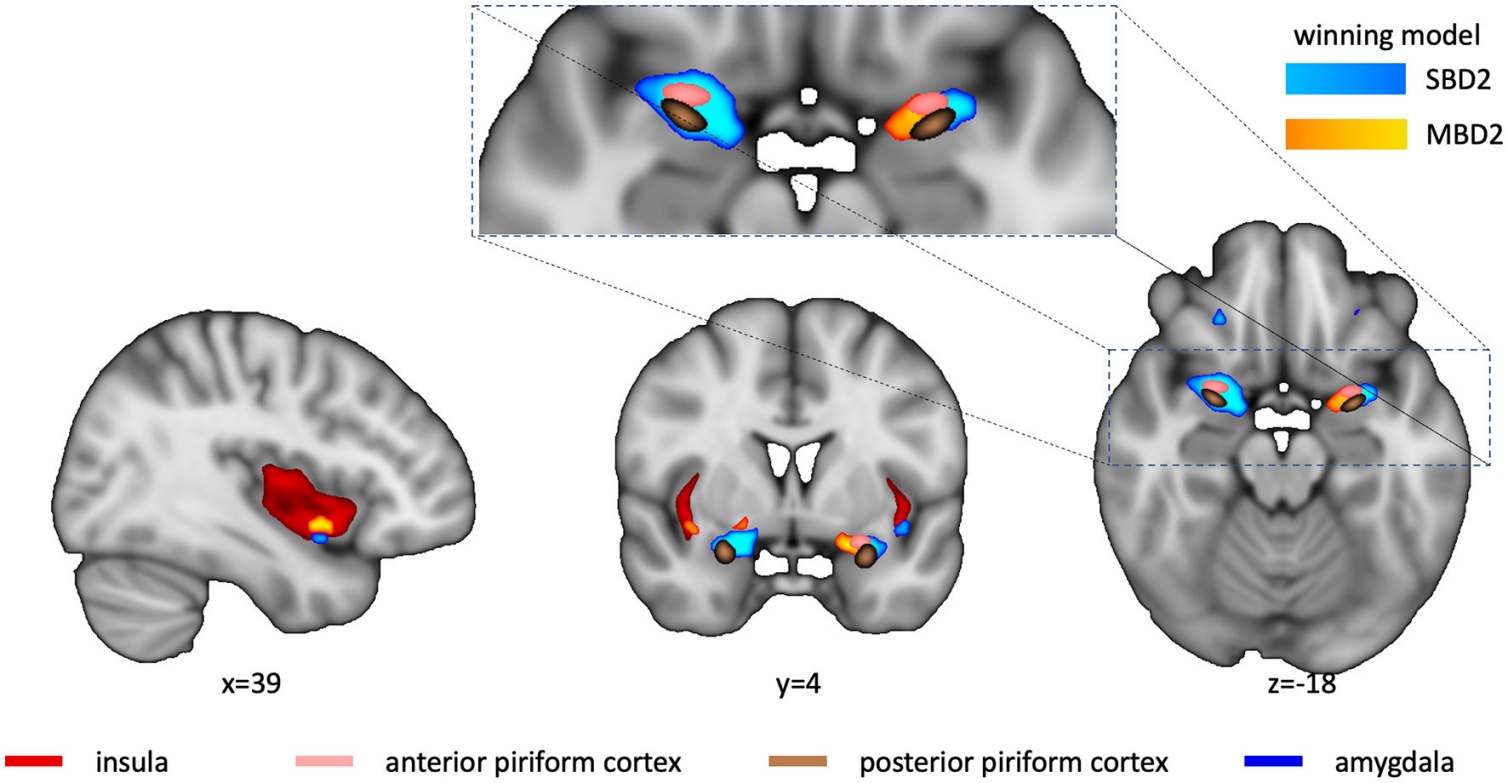
Winner map. Voxel colors denote the model with the maximum g-value for all four analysis types (light blue for SBD2, yellow for MBD2; SBD1 or MBD1 never turned out to be winning models). Winners were only computed for Hedge's *g* > 0.4. Anatomical ROIs (insula-red, anterior and posterior piriform cortex-copper, and amygdala-pink) are shown for reference. The cutout reveals that there were regional preferences for model SBD2 in the right piriform cortex, whereas MBD2 showed higher power in medial aspects of the left piriform cortex. The sagittal and coronal sections reveal that MBD2 yielded the highest power in the right insular cortex, with a small spot at the inferior border that preferred SBD2.

To assess whether there were finer spatial differences in sensitivity for different types of analyses, we computed whole-brain effect size maps for SBD1, SBD2, MBD1, and MBD2 using the MES toolbox (Hentschke and Stüttgen, 2011). Subsequently, we determined for each voxel which model yielded the highest effect size. Figure 4 shows the “winner map” across all four analysis types. Overall, voxels in which any of the models exceeded a threshold of *g* > 0.4 were best explained either by model SBD2 or MBD2, both of which contrast presenting an odorant with presenting odorless air. The right insular cortex and medial aspects of the left piriform cortex exhibited a preference for a breathing-modulated analysis approach.

### 3.2 Design efficiencies

**Figure 5:**
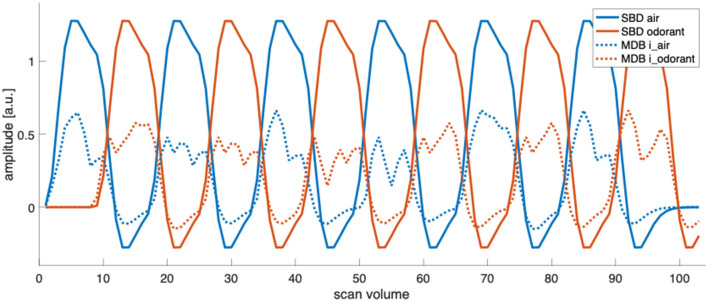
Predictor time courses for standard block designs (SBD) and one instance of inhalation-modulated block designs (MBD, for subject 2, run 1). In SBD1, design efficiency is exclusively determined by the variability (sum of squares) of the predictor “odorant”, and in SBD2, by the variability of the predictors “odorant” and “air” and their respective covariations. Similarly, in MBD1, design efficiency is exclusively determined by the variability (sum of squares) of the predictor “i_odorant”, and in MBD2, by the variability of the predictors “i_odorant” and “i_air” and their respective covariations (the computation of design efficiency is presented in Section 2.6). MBD predictors vary between participants and runs due to individual breathing patterns and fluctuations.

**Figure 6:**
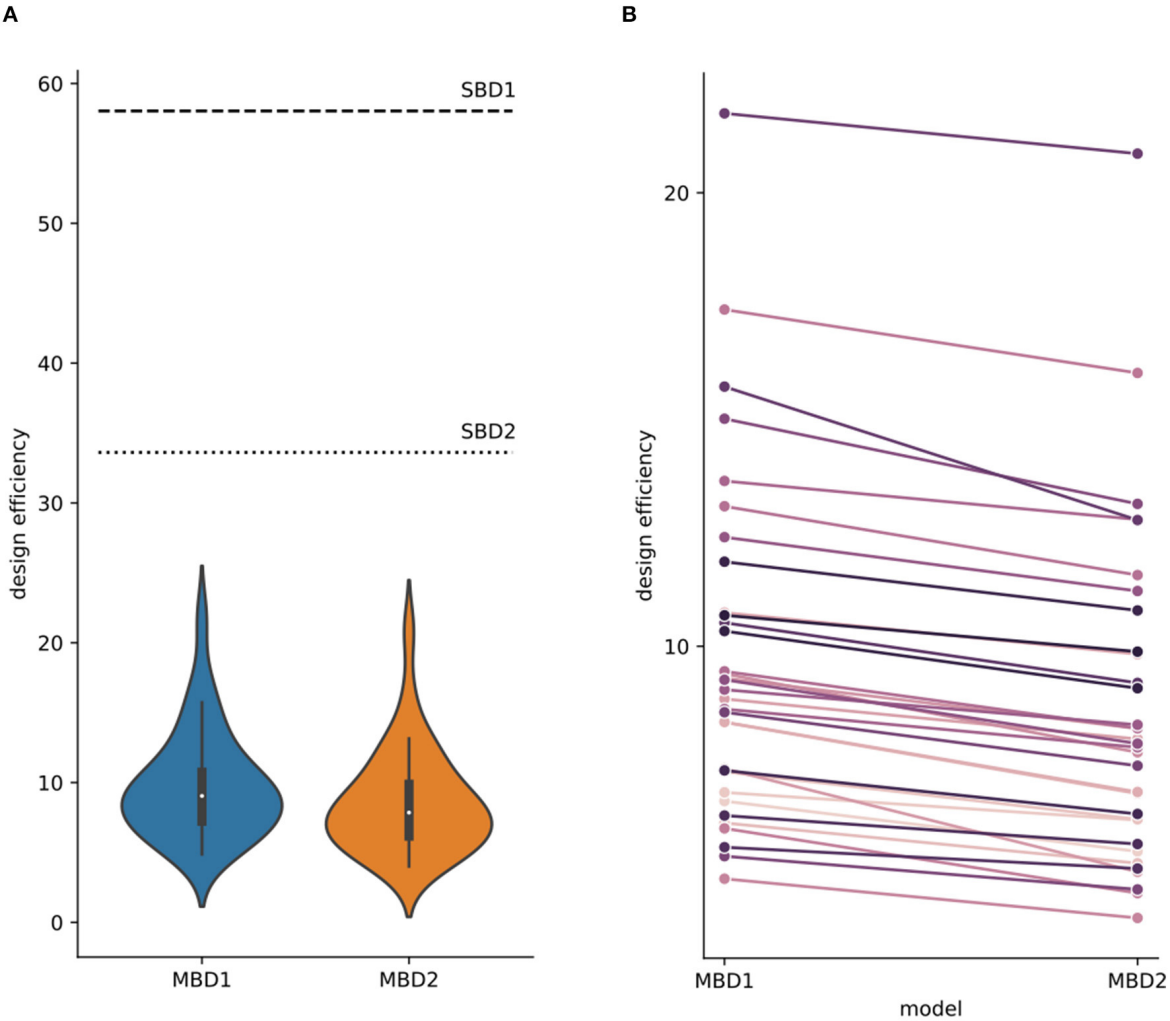
Design efficiencies for four different analysis strategies. **(A)** The design efficiency of the standard block designs (SBD1: horizontal dashed line; SBD2: horizontal dotted line) was only computed once because the timing of stimulation blocks was identical for all subjects and runs. Violin plots depict the design efficiencies for the breathing modulated block designs (MBD1: blue, MBD2: orange; the corresponding design matrices and contrasts are found in Figure 1D) for all subjects across runs. Design efficiencies differed between analysis strategies with efficiency_SBD1_ > efficiency_SBD2_ > efficiency_MBD1_ > efficiency_MBD2_ (Supplementary material S3 contains the statistical comparisons of design efficiencies). **(B)** In each participant, the average design efficiency (per subject across runs) was higher for MBD1 than for MBD2.

Design efficiency is a relative metric of how good a design is compared with other designs that have the same number of time points (Henson, 2007). Design efficiency increases with the design-induced variability of the predictors and decreases as more predictors co-vary. Figure 5 illustrates the predictor time courses for our four analysis approaches. SBD1, which relies only on the predictor for presenting an odorant, shows a design efficiency close to the maximally possible value given the amplitude and shape of the hemodynamic response function. MBD2, which relies on the predictors for inhalation-modulated odorant presentation (i_odorant) and inhalation-modulated non-odorant presentation (i_air), yields a substantially lower efficiency because the predictor amplitudes are substantially lower since the underlying inhalation events are much shorter than the accumulated block durations, and the variance of the predictors is lower. In both cases, SBD and MBD, adding a second predictor and the fact that the predictors show some covariance reduce the design efficiencies of SBD2 compared to SBD1 and of MBD2 compared to MBD1. The corresponding numerical results for the entire sample are depicted in Figure 6.

Modulating the blockwise predictors with inhalation events increased the design variance and therefore reduced the design efficiency. Figure 6 illustrates the design efficiencies of each single run for the four analysis strategies investigated here (SBD1-2, MBD1-2), yielding the order efficiencySBD1 (mean = 58.036) > efficiencySBD2 (mean = 33.614) > efficiencyMBD1 (mean: 9.747, std = 3.706) > efficiencyMBD2 (mean: 8.554, std = 3.575). The design efficiencies for SBD1 and SBD2 were constant for all runs in all subjects (dashed-dotted horizontal lines). Efficiency values are listed in Table 2.

**Table 2:** Design efficiencies for standard block designs (SBD1-2) and breathing-modulated block designs (MBD1-2).

	***N***	**Mean**	**Std. Deviation**
eff_SBD1_	-	58.036	-
eff_SBD2_	-	33.614	-
eff_MBD1_	33	9.747	3.706
eff_MBD2_	33	8.554	3.575

It should be noted that the design efficiency for SBD1 and SBD2 was constant for each subject.

### 3.3 Habituation

**Figure 7:**
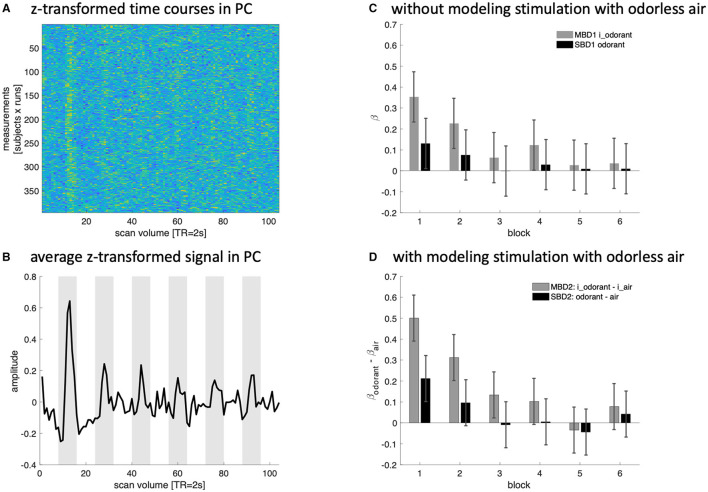
Z-transformed data **(A)** and a simple average time course **(B)** for the piriform cortex (PC) show a clear signal increase during the first block of presenting odorants, which quickly habituates with subsequent presentations. **(C, D)** Depict the amplitude estimates for the average time course data in the piriform cortex generated by our four analysis strategies.

In each experimental run, there were six blocks of continuous odorant presentation interspersed with 16s of presentation with odorless air. Since the odorants were kept constant in a given run, we investigated the degree of habituation in the piriform cortex and the extent to which such habituation was picked up by our four analysis strategies. Figure 7 shows the z-transformed BOLD signal of all subjects and runs (Figure 7A) and its respective group-averaged time course (Figure 7B). Figure 7C depicts the amplitude estimates yielded by MBD1 and SBD1 (which model only periods of stimulation with an odorant). Figure 7D depicts the differential amplitude estimates (odorant—air) yielded by MBD2 and SBD2 (which model periods of stimulation with an odorant and periods of presentation with odorless air). We compared our analysis strategies in a repeated-measures design with three within-subjects factors [(A) model type: without (1) or with (2) modeling odorless stimulation, (B) with (MBD) or without (SBD) inhalation-modulation regressors, and (C) presentation block (1–6)]. This design reflects the codes MBD1, SBD1, MBD2, and SBD2 used throughout the study and the block numbers used in Figure 7.

All four variants (MBD1, SBD1, MBD2, and SBD2) exhibited a decrease in BOLD signal as a function of block [*block*: *F*_(5, 160)_ = 3.52, *p* < 0.0048 also after Greenhouse-Geisser correction *p*_GG_ <0.0132; all interactions of *block* with *type, modulation*, or *type x modulation* where n.s. at a level of 0.05]. Inhalation-modulated predictors yielded higher amplitude estimates than non-modulated predictors [*modulation*: *F*_(1, 32)_ = 11.0, *p* < 0.0023] without showing interaction effects [*type x modulation*, *F*_(1, 32)_ = 3.18, *p* < 0.0839, n.s.]. Model type, i.e., including regressors for stimulating with odorless air, did not yield any differences in estimated BOLD amplitudes [*type*: *F*_(1, 32)_ = 0.016, *p* < 0.899].

## 4 Discussion

We measured the blood oxygenation level-dependent response in 33 healthy subjects in a 3T MRI scanner whom we exposed to 12 different odorants (one odorant per run) in a design in which six 16-s epochs of stimulation with the respective odorant alternated with 16-s epochs of stimulation with odorless air. We investigated the respective sensitivities and design efficiencies of four different analysis strategies (the definition of design matrices and statistical contrasts is presented in Figure 1) that either ignored (standard block designs 1–2) or accounted for (inhalation-modulated block designs 1–2) the participants' breathing patterns.

We found that all four analysis approaches were able to identify core areas of the human olfactory system, namely the bilateral piriform and orbitofrontal cortices, which is in good agreement with the existing literature (Gottfried, 2015; Torske et al., 2022). However, the four analysis approaches differed substantially in their ability to detect olfaction-related brain activity. SBD2 and MBD2, i.e., those designs that contrasted stimulation with an odorant with stimulation with odorless air, emerged as the most powerful designs (Figures 3, 4). The breathing-modulated design MBD2 [which modeled the BOLD time courses as a convolution of experimental blocks (odorant, no odorant), inhalation events (i), and a hemodynamic response function, and which contrasted the two resulting predictors (**i_odorant** vs. **i_air**), Figure 1] exhibited the highest power of all tested models in parts of the left piriform cortex and the right insula (Figure 4).

It should be noted that inhalation-modulated designs were comparable to, and in some parts of the brain, even more sensitive than, standard block design (SBD) analysis, despite having substantially lower detection power from a pure design perspective (Figures 5, 6). It should also be noted that design efficiency is a relative metric of how good a design is compared with other designs that have the same number of time points (Henson, 2007). From a purely mathematical standpoint, design efficiency is proportional to power, i.e., as design efficiency increases, power increases, unless there are differences in how well the different models reflect the underlying physiology.

This may seem surprising at first, but this finding points to the fact that assuming a box car function, i.e., a period of constant neural activity across blocks of constant stimulation with an odorant, is a poor reflection of the dynamics of olfaction, which consists of sampling events that are time-locked to sniffing or inhaling. Thus, taking such inhalation events into account proves to be highly beneficial despite the loss in design efficiency due to increased predictor variance (Friston et al., 1999). Georgiopoulos et al. (2018) observed that longer stimulation periods led to oscillatory signals, which they found hard to capture with a boxcar function, and therefore argued for short stimulation periods of 6 s for imaging the olfactory system. A similar finding and conclusion were reported by Schäfer et al. (2019), who found that 6-s stimulation blocks resulted in higher statistical sensitivity than longer block durations (also in Han et al., 2020). These studies used an analysis strategy akin to our SBD. We argue that breathing makes the olfactory signal oscillatory and is difficult to detect with such standard analyses of block designs that use a boxcar function over the entire stimulation block, but that such difficulties can be overcome by using inhalation-modulated block designs (our MBD designs) that model the sampling process of olfaction using the readily available information from a breathing belt.

Another benefit of MBD2, which may explain its slightly higher detection power than MBD1, is that MBD1 models the BOLD time courses with the predictor **i_odorant** only, thereby confounding olfaction and inhalation. It has been shown that inhalation alone can result in the activation of olfactory brain structures (Sobel et al., 1998). MBD2 instead models inhalation plus odorant (**i_odorant**) and inhalation of odorless air (**i_air**) and contrasts these two predictors. Following Donder's subtraction logic (Donders, 1969; Sternberg, 1969), this yields *(inhalation* + *odor processing) – inhalation* = *odor processing* if pure insertion applies, but a critique of the assumption of pure insertion is in Friston et al. (1996). Therefore, despite the further reduction in design efficiency due to increased predictor variance and correlated predictors (**i_odorant** and **i_air**), there is a benefit in the sensitivity of modeling and contrasting the two processes.

Presenting the same odorant repeatedly over the course of a run yielded substantial habituation effects in the piriform cortex (Figure 7), which is in line with previous findings in human (Sobel et al., 2000; Poellinger et al., 2001) and rat (Zhao et al., 2016) imaging. These habituation effects were picked up by all four of our analysis approaches (MBD1, SBD1, MBD2, and SBD2). Inhalation-modulated imaging appeared to yield higher estimates of BOLD amplitudes, but habituation results did not differ significantly from standard block designs. Similarly, including stimulation with odorless air in the model (MBD2, SBD2) did not lead to statistically different estimated habituation effects. Despite this statistical equivalence of the four analysis approaches, it appears that SBD models (SBD1 and 2) were unable to detect olfaction-induced BOLD signals after the first block (~4 sniffs of the same odorant), whereas the inhalation-modulated approaches started to fail after ~8 sniffs or two blocks (MBD 1) or ~12 sniffs or three blocks (MBD2), respectively.

### 4.1 Limitations

The BOLD signal in the primary olfactory cortex (POC) only persists for a few seconds (Sobel et al., 2000; Poellinger et al., 2001; Tabert et al., 2007). Furthermore, our findings are in agreement with reports that primary olfactory regions show substantial habituation with repeated presentation of the same odorant using electrophysiological methods in rats (Wilson, 1998) and neuroimaging in humans (Sobel et al., 2000). Because of such habituation, our design appears to be ill-suited to accurately estimate the shape of the hemodynamic response function, which could have improved the sensitivity of our inhalation-modulated models to detect odor-evoked BOLD responses. Here, we used a standard dual-gamma model that has been developed using responses of the early visual cortex to simple visual patterns (Glover, 1999), which also appears to provide a reasonable fit to olfactory responses in event-related paradigms (Anderson et al., 2003).

### 4.2 Outlook

In general, block-design paradigms have been considered relatively ineffective for olfactory fMRI studies (Wang et al., 2014), and an optimal strategy for investigating primary olfactory cortex most likely consists of rapid event-related designs that a) are timed using explicit instructions (“sniff now”) (Anderson et al., 2003) or using inhalation-triggered stimulus presentation^11^, and b) that switch between different odorants to avoid habituation.

However, depending on the scientific question, block designs with stimulation periods that extend beyond a few seconds may still be the optimal choice when trying to investigate mental states that either need time to build up or that are used as contextual factors that modulate ongoing cognitive and emotional processes. We expect that the latter designs will profit from the breathing-modulated analyses described here when it comes to separating sensory from higher-level processing.

## Data availability statement

The raw data supporting the conclusions of this article will be made available by the authors, without undue reservation.

## Ethics statement

The studies involving humans were approved by Ethikkommission der Universität Regensburg. The studies were conducted in accordance with the local legislation and institutional requirements. The participants provided their written informed consent to participate in this study.

## Author contributions

JS: Conceptualization, Data curation, Formal analysis, Funding acquisition, Investigation, Methodology, Project administration, Resources, Software, Supervision, Validation, Visualization, Writing — original draft, Writing — review & editing. A-LA: Formal analysis, Funding acquisition, Investigation, Project administration, Writing — original draft, Writing — review & editing. DT: Investigation, Writing — review & editing. SW: Investigation, Writing — review & editing. PS: Investigation, Writing — review & editing. MR: Investigation, Writing — review & editing. TH: Conceptualization, Resources, Writing — review & editing.
