# Supplementary material for: Inhalation-modulated detection of olfactory BOLD responses in the human brain
Source: Front Neuroimaging. 2023 Dec 1;2:1260893. doi: 10.3389/fnimg.2023.1260893 (PMC10725246; doi:10.3389/fnimg.2023.1260893)
Supplement: Supplementary file 1 [file Data_Sheet_1.docx]

Supplementary Material

Article Title

Aino-Lotta I. Alahäivälä, Divesh Thaploo, Simon Wein, Philipp Seidel, Marco Riebel, Thomas Hummel, Jens Volkmar Schwarzbach*

*** Correspondence:** Corresponding Author: jens.schwarzbach@ukr.de

[1 S1. Normalization of Respiratory Data 2](#_Toc150941719)

[2 S2. Preprocessing 4](#_Toc150941720)

[3 S3. Power and efficiency of fMRI designs 7](#_Toc150941721)

# S1. Normalization of Respiratory Data

Due to autocalibration of the physiological monitoring unit amplitudes in respiratory data could be subject to systematic changes over the course of a run. This can make identification with Matlab’s peak-finding algorithm difficult. Therefore, we submitted prefiltered z-transformed respiratory data to a standardization procedure (see eq. 1 and listing 1) to boost small signal amplitudes and to attenuate large amplitudes.

$y_{preprocessed}=\frac{y}{\left| y \right|^{\frac{1}{p}}}$ (eq. 1)

function y = enzo(y, n_iter)

p = 2;

for i = 1:n_iter

y = y./(abs(y).^(1/p));

end

*Listing 1. Iterative function enzo() that upscales small (|y| < 1) and downscales large (|y| > 1) signal amplitudes such that positive values are pushed towards +1 and negative values towards -1. n_iter = 4 yielded sufficient normalization for robust peak detection in the acquired respiratory data.*

**Error! Reference source not found.** shows the effect of this normalization procedure on to a sine wave with time varying amplitude, mimicking a putative recalibration of the PMU over time. *Figure S* ***2*** shows the effect of the same procedure on two examples of respiratory time course data (panel A, stable data; panel B data with decreasing amplitude over time). The normalization procedure leads to good detectability of inhalation-related peaks even in the presence of drift or recalibration.

*Figure S 1. Effect of applying the normalization procedure to a sine wave with time varying amplitude (original, blue). With each iteration, peaks are pushed towards +1 and troughs towards -1.*

| A S02, run 1   |
| --- |
| B S01, run 5   |

*Figure S 2. Effect of applying the normalization procedure to unproblematic respiratory data (subject 2, run 1, panel A) and to respiratory data that exhibited large amplitudes at the beginning and smaller amplitudes towards the end of the run (subject 1, run 5, panel B, blue line). Panel A shows that peak-information remains roughly unchanged. In panel B, the original respiratory data (blue line) shows different scaling over time. After applying the filter 3 times, peaks have an amplitude of about +1, and troughs an amplitude of about -1.*

# S2. Preprocessing

Results included in this manuscript come from preprocessing performed using *fMRIPrep* 20.2.4 (@fmriprep1; @fmriprep2; RRID:SCR_016216), which is based on *Nipype* 1.6.1 (@nipype1; @nipype2; RRID:SCR_002502).

Anatomical data preprocessing

: A total of 1 T1-weighted (T1w) images were found within the input BIDS dataset.The T1-weighted (T1w) image was corrected for intensity non-uniformity (INU) with `N4BiasFieldCorrection` [@n4], distributed with ANTs 2.3.3 [@ants, RRID:SCR_004757], and used as T1w-reference throughout the workflow.

The T1w-reference was then skull-stripped with a *Nipype* implementation of the `antsBrainExtraction.sh` workflow (from ANTs), using OASIS30ANTs as target template. Brain tissue segmentation of cerebrospinal fluid (CSF), white-matter (WM) and gray-matter (GM) was performed on the brain-extracted T1w using `fast` [FSL 5.0.9, RRID:SCR_002823, @fsl_fast].

Brain surfaces were reconstructed using `recon-all` [FreeSurfer 6.0.1, RRID:SCR_001847, @fs_reconall], and the brain mask estimated previously was refined with a custom variation of the method to reconcile ANTs-derived and FreeSurfer-derived segmentations of the cortical gray-matter of Mindboggle [RRID:SCR_002438, @mindboggle].

Volume-based spatial normalization to two standard spaces (MNI152NLin2009cAsym, MNI152NLin6Asym) was performed through nonlinear registration with `antsRegistration` (ANTs 2.3.3), using brain-extracted versions of both T1w reference and the T1w template.

The following templates were selected for spatial normalization:

*ICBM 152 Nonlinear Asymmetrical template version 2009c* [@mni152nlin2009casym, RRID:SCR_008796; TemplateFlow ID: MNI152NLin2009cAsym], *FSL's MNI ICBM 152 non-linear 6th Generation Asymmetric Average Brain Stereotaxic Registration Model* [@mni152nlin6asym, RRID:SCR_002823; TemplateFlow ID: MNI152NLin6Asym].

Functional data preprocessing

: For each of the 12 BOLD runs found per subject (across all tasks and sessions), the following preprocessing was performed. First, a reference volume and its skull-stripped version were generated using a custom methodology of *fMRIPrep*. A B0-nonuniformity map (or *fieldmap*) was estimated based on a phase-difference map calculated with a dual-echo GRE (gradient-recall echo) sequence, processed with a custom workflow of *SDCFlows* inspired by the [`epidewarp.fsl` script] (http://www.nmr.mgh.harvard.edu/~greve/fbirn/b0/epidewarp.fsl) and further improvements in HCP Pipelines [@hcppipelines]. The *fieldmap* was then co-registered to the target EPI (echo-planar imaging) reference run and converted to a displacements field map (amenable to registration tools such as ANTs) with FSL's `fugue` and other *SDCflows* tools. Based on the estimated susceptibility distortion, a corrected EPI (echo-planar imaging) reference was calculated for a more accurate co-registration with the anatomical reference.

The BOLD reference was then co-registered to the T1w reference using `bbregister` (FreeSurfer) which implements boundary-based registration [@bbr]. Co-registration was configured with six degrees of freedom. Head-motion parameters with respect to the BOLD reference (transformation matrices, and six corresponding rotation and translation parameters) are estimated before any spatiotemporal filtering using `mcflirt` [FSL 5.0.9, @mcflirt].

BOLD runs were slice-time corrected to 0.945s (0.5 of slice acquisition range 0s-1.89s) using `3dTshift` from AFNI 20160207 [@afni, RRID:SCR_005927]. The BOLD time-series were resampled onto the following surfaces (FreeSurfer reconstruction nomenclature): *fsaverage*. The BOLD time-series (including slice-timing correction when applied) were resampled onto their original, native space by applying a single, composite transform to correct for head-motion and susceptibility distortions. These resampled BOLD time-series will be referred to as *preprocessed BOLD in original space*, or just *preprocessed BOLD*.

The BOLD time-series were resampled into standard space, generating a *preprocessed BOLD run in MNI152NLin2009cAsym space*. First, a reference volume and its skull-stripped version were generated using a custom methodology of *fMRIPrep*. *Grayordinates* files [@hcppipelines] containing 91k samples were also generated using the highest-resolution ``fsaverage`` as intermediate standardized surface space.

Several confounding time-series were calculated based on the *preprocessed BOLD*: framewise displacement (FD), DVARS and three region-wise global signals. FD was computed using two formulations following Power (absolute sum of relative motions, @power_fd_dvars) and Jenkinson (relative root mean square displacement between affines, @mcflirt). FD and DVARS are calculated for each functional run, both using their implementations in *Nipype* [following the definitions by @power_fd_dvars]. The three global signals are extracted within the CSF, the WM, and the whole-brain masks. Additionally, a set of physiological regressors were extracted to allow for component-based noise correction [*CompCor*, @compcor]. Principal components are estimated after high-pass filtering the *preprocessed BOLD* time-series (using a discrete cosine filter with 128s cut-off) for the two *CompCor* variants: temporal (tCompCor) and anatomical (aCompCor). tCompCor components are then calculated from the top 2% variable voxels within the brain mask. For aCompCor, three probabilistic masks (CSF, WM and combined CSF+WM) are generated in anatomical space. The implementation differs from that of Behzadi et al. in that instead of eroding the masks by 2 pixels on BOLD space, the aCompCor masks are subtracted a mask of pixels that likely contain a volume fraction of GM. This mask is obtained by dilating a GM mask extracted from the FreeSurfer's *aseg* segmentation, and it ensures components are not extracted from voxels containing a minimal fraction of GM. Finally, these masks are resampled into BOLD space and binarized by thresholding at 0.99 (as in the original implementation). Components are also calculated separately within the WM and CSF masks. For each CompCor decomposition, the *k* components with the largest singular values are retained, such that the retained components' time series are sufficient to explain 50 percent of variance across the nuisance mask (CSF, WM, combined, or temporal). The remaining components are dropped from consideration.

The head-motion estimates calculated in the correction step were also placed within the corresponding confounds file. The confound time series derived from head motion estimates and global signals were expanded with the inclusion of temporal derivatives and quadratic terms for each [@confounds_satterthwaite_2013]. Frames that exceeded a threshold of 0.5 mm FD or 1.5 standardised DVARS were annotated as motion outliers.

All resamplings can be performed with *a single interpolation step* by composing all the pertinent transformations (i.e., head-motion transform matrices, susceptibility distortion correction when available, and co-registrations to anatomical and output spaces). Gridded (volumetric) resamplings were performed using `antsApplyTransforms` (ANTs), configured with Lanczos interpolation to minimize the smoothing effects of other kernels [@lanczos]. Non-gridded (surface) resamplings were performed using `mri_vol2surf` (FreeSurfer).

Many internal operations of *fMRIPrep* use *Nilearn* 0.6.2 [@nilearn, RRID:SCR_001362], mostly within the functional processing workflow. For more details of the pipeline, see [the section corresponding to workflows in *fMRIPrep*'s documentation] (https://fmriprep.readthedocs.io/en/latest/workflows.html "FMRIPrep's documentation").

### Copyright Waiver

The above boilerplate text was automatically generated by fMRIPrep with the express intention that users should copy and paste this text into their manuscripts *unchanged*.

It is released under the [CC0](https://creativecommons.org/publicdomain/zero/1.0/) license.

# S3. Power and efficiency of fMRI designs

One-Sample Test efficiency MB-designs vs. efficiency of SBD

A) Test Value = 58.0375 (efficiency of SBD1)

|  |  |  |  |  | 95% Confidence Interval of the Difference | |
| --- | --- | --- | --- | --- | --- | --- |
|  | t | df | p  (2-tailed) | Mean Difference | Lower | Upper |
| eff_MBD1_ | -73.717 | 32 | < .00001 | -48.290 | -49.625 | -46.956 |
| eff_MBD2_ | -78.300 | 32 | < .00001 | -49.483 | -50.771 | -48.196 |

B) Test Value =33.614 (efficiency of SBD2)

|  |  |  |  |  | 95% Confidence Interval of the Difference | |
| --- | --- | --- | --- | --- | --- | --- |
|  | t | df | p  (2-tailed) | Mean Difference | Lower | Upper |
| eff_MBD1_ | -36.433 | 32 | < .00001 | -23.867 | -25.201 | -22.534 |
| eff_MBD2_ | -39.654 | 32 | < .00001 | -25.060 | -26.347 | -23.773 |

*Table S1. A, B) Comparisons of design efficiencies for breathing-modulated block designs (MBD1-2) with the design efficiency of the standard block designs (SBD1-2) by means of one-sample t-tests. MBD1 and MBD 2 did not differ statistically (see Figure 6 in the main manuscript).*
